# Preparation of Tween 80-Zn/Al-Levodopa-Layered Double Hydroxides Nanocomposite for Drug Delivery System

**DOI:** 10.1155/2014/104246

**Published:** 2014-03-23

**Authors:** Aminu Umar Kura, Samer Hasan Hussein-Al-Ali, Mohd Zobir Hussein, Sharida Fakurazi

**Affiliations:** ^1^Laboratory of Vaccine and Immunotherapeutic, Institute of Bioscience, Universiti Putra Malaysia, 43400 Selangor, Malaysia; ^2^Laboratory of Molecular Biomedicine, Institute of Bioscience, Universiti Putra Malaysia, 43400 Selangor, Malaysia; ^3^Faculty of pharmacy, Isra university, P.O. Box 22, Amman 11622, Jordan; ^4^Materials Synthesis and Characterization Laboratory, Institute of Advanced Technology (ITMA), Universiti Putra Malaysia, 43400 Selangor, Malaysia; ^5^Faculty of Medicine and Health Science, Pharmacology Unit, Universiti Putra Malaysia, 43400 Selangor, Malaysia

## Abstract

We incorporated anti-Parkinsonian drug, levodopa (dopa), in Zn/Al-LDH by coprecipitation method to form dopa-LDH nanocomposite. Further coating of Tween-80 on the external surfaces of dopa-LDH nanocomposite was achieved through the oxygen of C=O group of Tween-80 with the layer of dopa-LDH nanocomposite. The final product is called Tween-dopa-LDH nanocomposite. The X-ray diffraction indicates that the Tween-dopa-LDH nanocomposite was formed by aggregation structure. From the TGA data, the Tween-80 loading on the surface of LDH and dopa-LDH was 8.6 and 7.4%, respectively. The effect of coating process on the dopa release from Tween-dopa-LDH nanocomposite was also studied. The release from Tween-dopa-LDH nanocomposite shows slower release compared to the release of the drug from dopa-LDH nanocomposite as done previously in our study, presumably due to the retarding shielding effect. The cell viability study using PC12 showed improved viability with Tween-80 coating on dopa-LDH nanocomposite as studied by mitochondrial dehydrogenase activity (MTT assay).

## 1. Introduction

Nanotechnology is gaining more acceptability in the area of biomedical sciences especially in the field of drug delivery due to increase precision in specific tissue drug delivery and decrease toxicity among other advantages compared to conventional drug delivery system [1]. Layered double hydroxides (LDHs) are one of nanolayer materials used in drug delivery; its synthesis is relatively easy in the laboratory for drug delivery, usually with controllable size and versatile compositions. It has a very good biocompatibility as well as low cytotoxicity profile, giving perfect cover to the intercalated drug by virtue of its interlayer structure [2]. However, poor water solubilisation, agglomeration, inadequate targeted delivery, minimal thermal stability, and surface charges of nanoparticles (LDHs inclusive) intended for drug delivery may be a hindrance [3]. Significant influence in the physicochemical and pharmaceutical properties of the nanoparticles through surface modification with surfactants or polymer or both can alter particle size, size distribution, particles morphology, surface chemistry, surface hydrophobicity, zeta potential, and drug encapsulation efficiency. These will likely improve the water solubility of a nanoparticles, decrease agglomeration, and lead to a more targeted delivery of the nanoparticles with minimal toxicity [4]. Surface interaction and adherence of nanoparticles is significantly different compared to submicron sized particles as such nanoparticles have an extremely high tendency of adhesion and aggregation [3]. The choice of surface coating material is important and very critical for a successful nanoparticle formulation in drug delivery and other biomedical applications. For example, synthesized uncoated iron oxide nanoparticles were shown to aggregate in biological solutions due to their large surface area to volume ratio, forming large clusters and rendering them unsuitable for biomedical applications [5]. A number of materials have been used in the past as surface coating including dextran, polyethylene glycol (PEG), phospholipids, polyethyleneimine (PEI), and most recently, chitosan [5]. These coating are sometimes used as targeting material, helping in nanoparticle delivery to specific areas, increasing the stability, and/or decreasing the toxicity potentials.

In the past, the synthesis of polymer-coated LDH has been reported [6, 7], either for targeted drug delivery and/or increasing stability. Several methods were used in the coating process, among which are reconstruction, ionic exchange, and spontaneous self-assembly method [6]. Unlike ion exchange and reconstruction method, spontaneous self-assembly produces dextran coated LDH maintaining the initial size and crystalline structure base on XRD result [7]. Maintaining the hydrotalcite-like compounds structure of LDH with positively charged metal oxide/hydroxide sheets intercalated with anions and water molecules is of essence in drug delivery via ion exchange [7].

However, in nanoparticle delivery to brain, Tween 80 appeared to be very helpful in mediating transport across the blood brain barrier (BBB) [8]. Apolipoprotein E adsorption by the surface of Tween 80 coated nanoparticles is believed to be responsible for this transport. Particles attached with this plasma protein seem to mimic low density lipoprotein (LDL) whose receptors are in abundant in the endothelial cells lining the BBB leading to their uptake into the brain [8].

Hence, the choice of Tween-80 surfactant as coating material in this study for levodopa delivery to the brain is timely. Here, we aimed at studying the stability, realizing ability, and toxicity profile of LDHs intercalated with levodopa and coated with Tween 80.

## 2. Experimental Procedures

### 2.1. Materials

L-3-(3,4-dihydroxyphenyl) alanine (levodopa, 99% purity), Tween-80, and 3-(4,5-dimethylthiazol-2-yl)-2,5-diphenyltetrazolium bromide (MTT) were purchased from Sigma-Aldrich (St. Louis, MO, USA). Other chemicals, including zinc nitrate hexahydrate (Zn(NO_3_)_2_·6H_2_O) and aluminum nitrate nonahydrate (Al(NO_3_)_3_·9H_2_O), were of analytical grade and used without further characterization. Deionized water was used throughout the experiment.

### 2.2. Synthesis

Zn/Al-LDH was synthesized by adding a solution of 1 Mol/L sodium hydroxide dropwise to a mixture solution of zinc and aluminum nitrate, with initial molar ratio 2 : 1, in deionized water under a nitrogen atmosphere while vigorously stirring until a pH of 7.0 was reached. The mixture was aged in an oil bath for 18 hours at 70°C. The white precipitate obtained was centrifuged, washed three times with deionized water, and dried in an oven overnight at 70°C. The product was denoted as Zn/Al-LDH.

Dopa-Zn/LDH nanocomposite was synthesized as described in our previous work using a direct coprecipitation method [9]. In brief, a solution of levodopa (0.08 molar) was added to a Zn(NO_3_)_2_·6H_2_O and Al(NO_3_)_3_·9H_2_O solutions, at a ratio of 2 : 1, under constant stirring in the presence of a sustained nitrogen supply at room temperature, and the pH was adjusted to 7.0 using 1.0 molar NaOH. The experiment was protected from direct sunlight exposure because of the sensitivity of levodopa to light. The mixture was aged at 70°C in an oil bath for 18 hours and then centrifuged, filtered, washed with deionized water three times, and dried in an oven overnight. The product was denoted as dopa-LDH nanocomposite (levodopa intercalated into Zn/Al-LDH).

Coated dopa-LDH nanocomposite by Tween-80 was synthesized according to literature [10]. Dopa-LDH nanocomposite (0.2 g) was added to Tween-80 dissolved solution (5 mM and 100 mL) and reacted with stirring for 18 hour. The precipitate was washed with deionized water three times and dried in an oven over night. The product was denoting as Tween-dopa-LDH nanocomposite (Tween-80 coated dopa-LDH nanocomposite). The coated Zn/Al-LDH by Tween-80 was synthesized similar to Tween-dopa-LDH nanocomposite and the final product was denoting as Tween-LDH.

### 2.3. Controlled-Release Study

Levodopa release profiles from Tween-dopa-LDH nanocomposite were determined at room temperature using phosphate-buffered saline at pH 4.8 and 7.4. Approximately 300 mg of the nanocomposite was added to 500 mL of the medium. The accumulated amount of levodopa released into the solution was measured at preset time intervals and at *λ*_max⁡_ = 280 nm using an ultraviolet-visible spectrophotometer (Lambda 35, Perkin-Elmer, Boston, MA, USA).

### 2.4. Cell Culture and Treatment

A dopaminergic cell model (PC12) was obtained from American Type Culture Collection (ATCC) Manassas, VA. USA. To maintain the cell line we used RPMI 1640D medium enriched with 10% fetal bovine serum (FBS), 15 mmol/L L-glutamine, 100 units/mL penicillin, and 100 *μ*g/mL streptomycin, cells were kept in a humidified incubator at 37°C and 5% CO_2_. Cells were seeded into 96-well plate at 1 × 10^5^ cells/mL and kept overnight for cells attachment.

### 2.5. Preparation of Nanocomposite for Viability Assay

Dopa-LDH nanocomposites as well as the Zn/Al-LDH carrier were dispersed in PBS. A stock suspension of 10 mg/mL of each nanocomposite was made by sonication for 5 minutes and culture medium was used to obtain the desired concentration via serial dilution. The nanocomposite were further dispersed through vortex agitation for 2 mins before usage. A dose range 25 *μ*/mL to 800 *μ*g/mL used and cells were exposed for 72 hours to assess the impact of our treatment on their viability for toxicity effects. Well contained cells and media only were used as control to compare. Experiment was done in triplicate and each time a freshly prepared nanoparticle is used to treat cells and results were presented as mean ± SD.

### 2.6. Cell Viability Study

MTT assay was used to assess the viability of PC 12 after exposure to different doses of the synthesized nanoparticles. In this assay, the MTT reagent (3,(4,5-dimethylthiazol-2-yl)-2,5-diphenyltetrazolium bromide) is converted to an insoluble and brightly coloured formazan by viable cells in the culture which correlate with the reading taken for viable cells [11]. It takes 2–4 hours for this conversion to happen and a detergent (DMSO) is then added to the cells to stop the conversion and solubilize the formazan. The plate was shaken in the dark for thirty to sixty minutes and absorbance of the formed formazan is taken at a wavelength of 570 nm using a suitable multiwall microplate reader. In brief, 20 *μ*L of MTT solution (5 mg/mL in PBS) was added to each well and kept in an incubator for 2 hour. The MTT-containing medium was removed gently and replaced with dimethyl sulfoxide (100 *μ*L/well) to mix with the formazan crystals until dissolved. Absorbance at 570 nm and 630 nm (background) was measured with a microplate Elisa reader (ELx800 from BioTek Instruments):

(1) $$\begin{array}{c} \%\text{Cytotoxicity}=\frac{\text{Average}\;\;\text{of}\;\;\text{treated}}{\text{Average}\;\;\text{control}}\times 100. \end{array}$$

### 2.7. Measurement of Homovalinic Acid by ELISA

An* in vitro* drug metabolism using the neurogenic PC-12 cells was used to study possible delivery of levodopa and its metabolism by measuring the drug end product called homovalinic acid (HVA) from treated cells. Monoamine oxidases (MAOs) A and B are the enzymes present in PC 12 cells and capable of deamination of biogenic amines including dopamine from levodopa into HVA [12]. RPMI-1640 media containing nerve growth factor (NGF) (NGF-7S, Sigma-Aldrich, Inc., St Louis, MO) at 100 ng/mL was used to differentiate the cells for this purpose as reported earlier [1]. We chose doses from the viability assay that allow for more than eighty percent cell vitality. Zero concentration used as control from which we measured the endogenously produce HVA from the neuronal cells. Cell lysate obtained by mechanical disruption of the treated and control cells was centrifuged at 15000 rpm for 15 minutes at 40°C.The supernatant obtained was used to detect HVA level, the procedure was base on manufacturers instruction contained in the Kit. For this study, we use CUSABIO Rat homovalinic acid (HVA) Elisa kit (Wuhan, Hubei Province 430206, China).

### 2.8. Characterization

Powder X-ray diffraction patterns were recorded in the 2°–70° range on a diffractometer (XRD-6000, Shimadzu, Tokyo, Japan) using CuK_*α*_ radiation (*λ* = 1.5418 Å) at 30 kV and 30 mA, with a dwell time of 4 degrees per minute. The Fourier transform infrared spectra of the materials were recorded at 400–4000 cm^−1^ using a Thermo Nicolet Nexus FTIR (model Smart Orbit) (International Equipment Trading Ltd., Vernon Hills, IL, USA). Thermogravimetric and differential thermogravimetric analyses (Mettler Toledo, Columbus, OH, USA) were carried out at a heating rate of 10°C per minute from 20 to 1000°C under a nitrogen atmosphere (N_2_ flow rate of 50 mL per minute). A field emission scanning electron microscope (Nova NanoSEM 230, FEI Company, Hillsboro, OR, USA) was used to determine the surface morphology of the samples.

## 3. Results and Discussion

### 3.1. Powder X-Ray Diffraction

**Figure 1:**
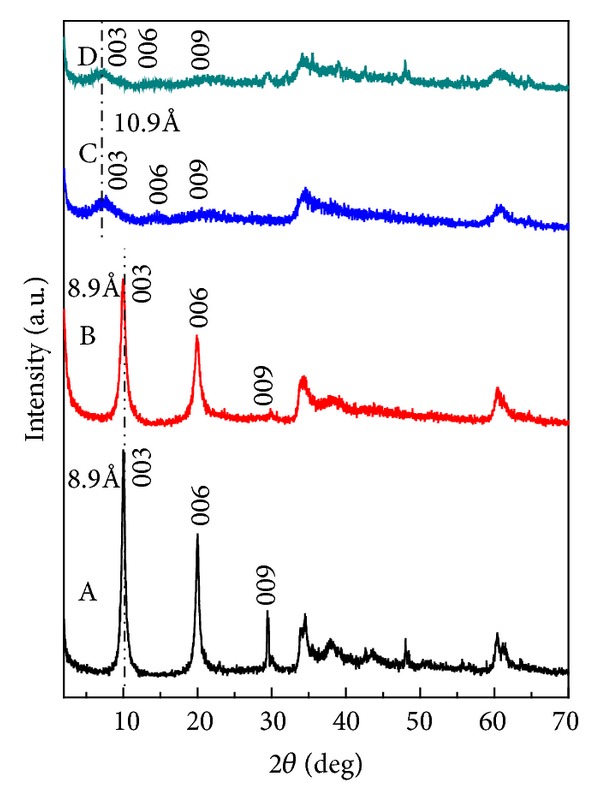
Powder X-ray diffraction patterns for the (A) Zn/Al-LDH, (B) Tween-LDH, (C) dopa-LDH nanocomposite, and (D) Tween-dopa-LDH nanocomposite.

The XRD patterns of the Zn/Al-LDH and dopa-LDH nanocomposite samples synthesized by coprecipitation method are shown in Figures 1(A) and 1(C), respectively. The (0 0 1) basal reflections of the synthesized dopa-LDH nanocomposite sample was shifted to lower angle side with broadening of the reflections, thus, the obtained d-spacing of (0 0 3) reflections were 10.9 Å and larger than 8.9 Å of Zn/Al-LDH peak. This result indicates that the dopa molecule was intercalated into the interlayers of LDHs.

The XRD patterns of Tween-80 after the coating of Zn/Al-LDH and dopa-LDH nanocomposite are shown in Figures 1(B) and 1(D), respectively. The Figure does not show any change between those of dopa-LDH nanocomposite (Figure 1(D)) and Tween-dopa-LDH nanocomposite (Figure 1(C)). This is because the additional Tween-80 molecules into the sample resulted in the adsorption of the former on the surfaces of LDH particles and were not exchanged with dopa intercalated into the interlayer of dopa-LDH nanocomposite.

**Figure 2:**
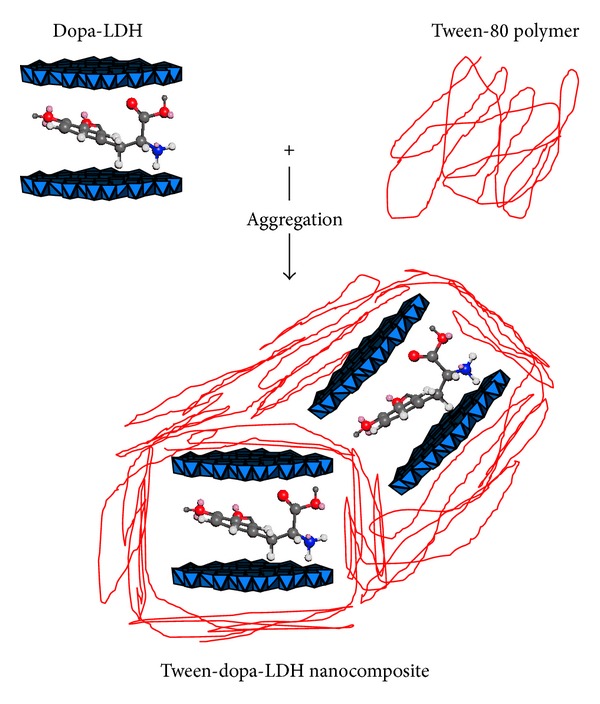
Schematic representation of the aggregation type of nanocomposite produce from the interaction between dopa-LDH nanocomposite and Tween-80 polymer.

Generally, polymer-LDH nanocomposite can be obtained from the association of LDH with polymers by different ways. The first type is called intercalated nanocomposite, where the polymeric chains were encapsulated into the interlayers of LDH and in this case, the interlayer spacing could be increased. The exfoliated nanocomposite is the second type, in which the materials present no ordering along the stacking axis of the layer. The third type is called aggregation, where there is no expansion of the basal spacing in the XRD; diffraction peaks can be observed. In the Tween-dopa-LDH nanocomposite, the sample will follow the last type (Figure 2) and the XRD diffraction peaks confirm that.

### 3.2. Infrared Spectroscopy

**Figure 3:**
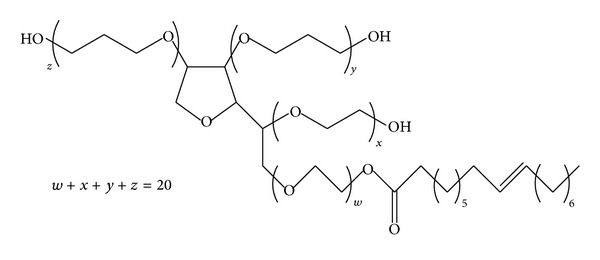
Structure of Tween-80.

**Figure 4:**
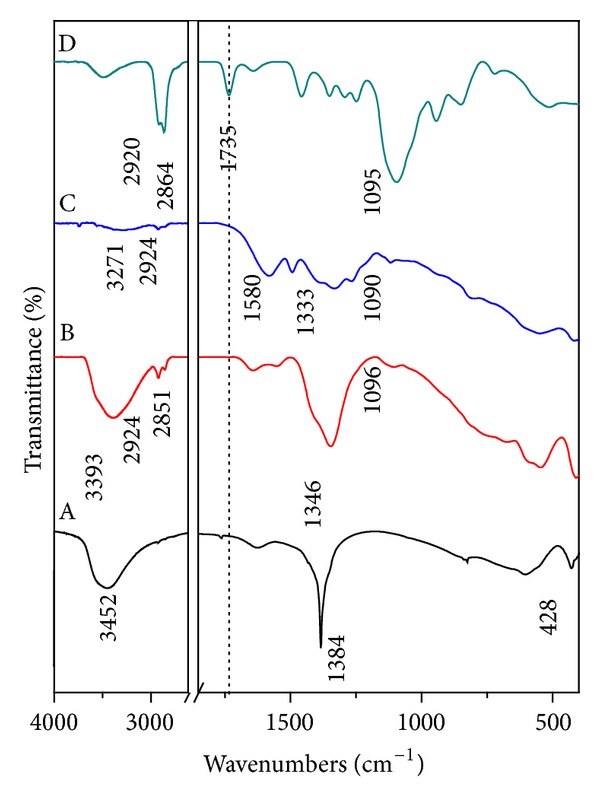
Fourier transform infrared spectra of Zn/Al-LDH (A), Tween-LDH (B), Tween-dopa-LDH nanocomposite (C), and free Tween-80 (D).

The FT-IR spectrum of Tween-80 is shown in Figure 4(D). Tween-80 shows many intense, sharp absorption peaks that are due to the different functional groups present in the molecules (Figure 3). Methyl group (–CH_3_) shows absorption band at 2920 cm^−1^, while the band at 2864 cm^−1^ is due to –CH_2_-stretching. The band at 1735 cm^−1^ can be attributed to C=O and the band at 1095 cm^−1^ is due to stretching of C–O–C [13].

Figure 4(A) shows the FTIR for Zn/Al-LDH. The absorption band at 1384 cm^−1^ was due to the stretching vibration of nitrate groups. A broad absorption band at 3452 cm^−1^ was attributed to stretching of O–H group, which is in the layer. While the absorption at 428 cm^−1^ is due to Zn-Al-OH stretching.

Figures 4(B) and 4(C) show the FT-IR spectra of Tween-LDH and Tween-dopa-LDH nanocomposite, respectively. The presence of most of the bands from the surfactant Tween 80 in the FT-IR spectrum of these samples confirms the presence of Tween-80 on the surface of Zn/Al-LDH and dopa nanocomposite.

The Tween-LDH gives absorption bands at 2924, 2851, and 1096 cm^−1^ which is related to –CH_3_, –CH_2_-, and C–O–C of Tween-80, respectively; whereas, the Tween-dopa-LDH nanocomposite shows bands at 2924, 2855, and 1090 cm^−1^ for the same functional groups. These results indicate the coating of the Zn/Al-LDH and dopa nanocomposite by the Tween-80. The absorption bands due to C=O stretching at 1735 cm^−1^ in Tween-80, however, cannot be observed in the surface modified-Zn/Al-LDH and dopa nanocomposite which might be due to the chemical interaction of Tween-80 with the surface of LDH via the oxygen of C=O group [13].

Figure 4(C) shows also different absorption peaks related to the dopa molecules, which indicate that the coating process was not affected on the intercalated dopa molecules [9].

### 3.3. Thermal Analysis

**Figure 5:**
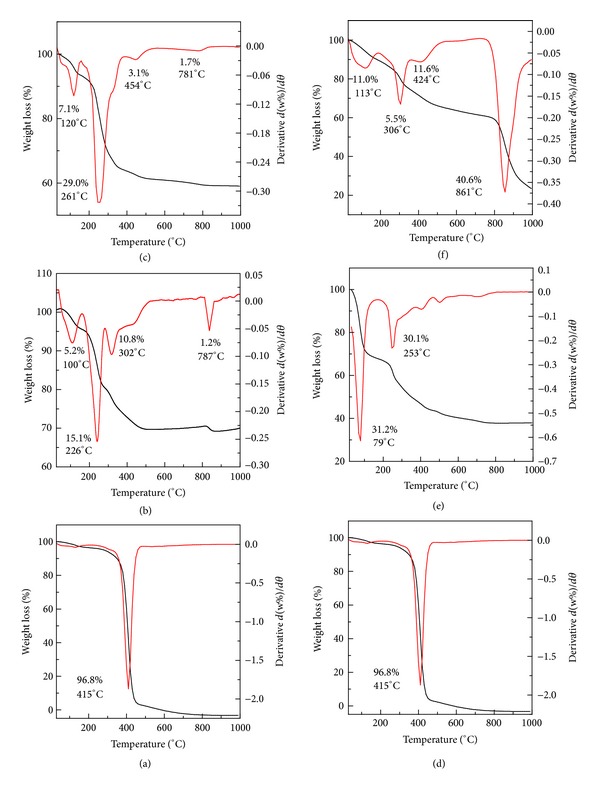
TGA/DTG thermograms of levodopa ((a) and (d)), Zn/Al-LDH (b), tween-LDH (c), dopa-LDH nanocomposite (e), and the tween-dopa-LDH-nanocomposite (f).

Thermal behavior of Zn/Al-LDH and dopa-LDH nanocomposite before and after coating by Tween-80 was examined using thermogravimetric and differential thermogravimetric analyses (Figure 5). For Tween-80 (Figures 5(a) and 5(d)), one main thermal event was clearly observed which occurred in the region of 240–480°C and was attributed to the combustion of Tween-80, corresponding to a sharp peak in the differential thermogravimetric curve at 415°C with 96.8% weight loss [14]. Figure 5(b) shows that the thermal decomposition of Zn/Al-LDH progressed through four major steps of weight loss at 100°C, 226°C, 302°C, and 787°C, with total weight losses of 32.3% [15]. Figure 5(c) shows the thermal behavior of Tween-LDH with four stages of weight loss and total weight loss of 40.9%. This result indicates that Tween-80 loading on the surface of Zn/Al-LDH is around 8.6%.

The thermal decomposition characteristics of the dopa-LDH nanocomposite are shown in Figure 5(e). The thermal decomposition was characterized by two weight loss events, one at 35°C–162°C with a weight loss of 31.2%, due to the removal of the external surface-adsorbed and interlayer water molecules, and a second event at 162°C–960°C with a weight loss of 30.1%. The second weight loss was due to dehydroxylation of the layers and decomposition of levodopa [9]. Figure 5(f) shows the thermal properties of tween-dopa-LDH nanocomposite. After the coating process, the thermal decomposition characteristics of the tween-dopa-LDH nanocomposite were similar to the precursor. The difference was only in the total weight loss, where the total weight loss in case of tween-dopa-LDH nanocomposite was 68.7%, compared to 61.3% for precursor of dopa nanocomposite. This result indicates that the dopa-LDH was coated with 7.4% Tween-80.

### 3.4. Surface Morphology

**Figure 6:**
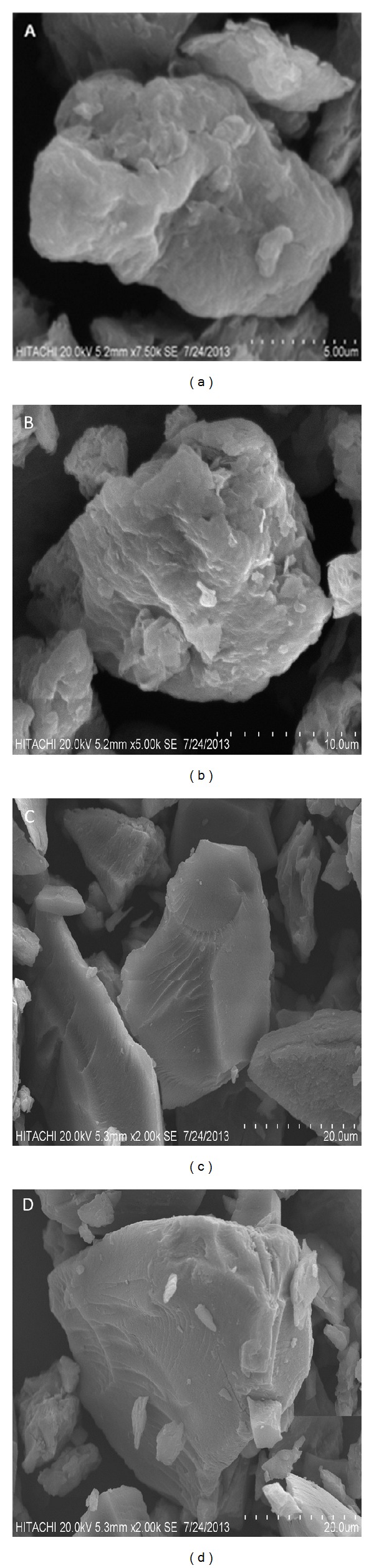
Field emission scanning electron micrographs of Tween-LDH ((a) and (b)), and of the tween-dopa-LDH nanocomposite ((c) and (d)).

The surface morphologies of Tween-LDH and coated dopa nanocomposite were illustrated in Figure 6. The Zn/Al-LDH and the dopa-LDH nanocomposite showed nonuniform, irregular agglomerates with compact and nonporous plate-like structures [9]. Tween-LDH and Tween-dopa-LDH nanocomposite show the same surface morphology with uncoated Zn/Al-LDH and the dopa-LDH nanocomposite with more agglomeration.

### 3.5. Release Behavior of Dopa from the Tween-Dopa-LDH Nanocomposite

**Figure 7:**
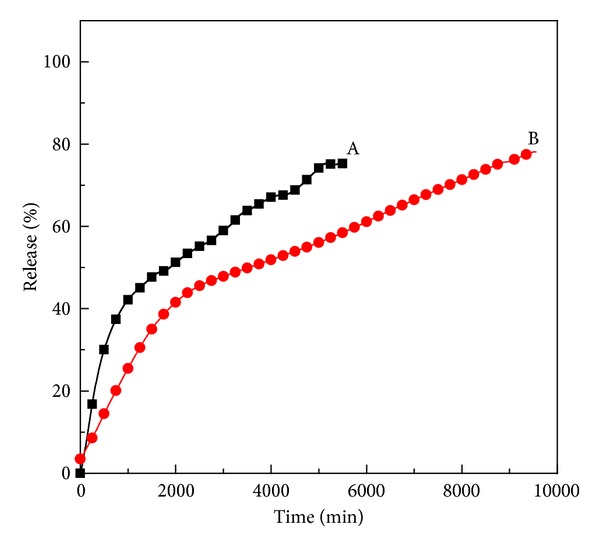
Release profiles of dopa from the Tween-dopa-LDH nanocomposite at pH 4.8 (A) and pH 7.4 (B).

Release profiles of dopa from tween-dopa-LDH nanocomposite were determined at constant temperature (25 ± 0.5°C) using PBS solution at pH 7.4 and 4.8 and the results are shown in Figure 6. The release behavior at pH 4.8 (Figure 7(A)) is faster compared to pH 7.4 (Figure 7(B)), which can be attributed to the partial dissolution of LDH layer under acidic environment [16, 17] and ion-exchange process between the intercalated anions in interlayer and phosphate anions in the buffer solution. The release step was characterized as released percentage of 75% after 5550 minute. At pH 7.4, the release of dopa is slower and persistent, and 78% of released percentage is obtained after 9550 minute. This slow and sustained release process may also be interpreted on the basis of the ion-exchange process between the dopa anions and phosphate anions in the buffer solution [17, 18]. In our previous report of controlled release of dopa from uncoated nanocomposite, release was reported to be 76% after 2400 and 8600 minutes at pH 4.8 and 7.4, respectively [9]. This difference is attributed to the retarding effect which results from Tween-80 molecules which was adsorbed and coated nanocomposite [10].

### 3.6. Release Kinetics of Levodopa from the Tween-Dopa-LDH Nanocomposite

Data obtained from* in vitro* release studies were fitted to various kinetic equations to find out the mechanism of dopa release from the coated nanocomposite. The kinetic models used were [19, 20].

 Pseudo-first-order:
  (2) $$\begin{array}{c} \ln\left(q_{e}-q_{t}\right)=\ln q_{e}-k_{1}t. \end{array}$$
 Pseudo-second-order:
  (3) $$\begin{array}{c} \frac{t}{q_{t}}=\frac{1}{k_{2}q_{e}^{2}}+\frac{t}{q_{e}}. \end{array}$$
 Parabolic diffusion:
  (4) $$\begin{array}{c} \frac{\left(1-M_{t}/M_{o}\right)}{t}=k_{d}^{-0.5}+b, \end{array}$$

where *q*_*e*_ and *q*_*t*_ are the equilibrium release amount and the release amount at any time (*t*), respectively, *M*_*o*_ and *M*_*t*_ are the initial amount and the amount of the drug in the nanocomposite at time *t*, respectively.

**Figure 8:**
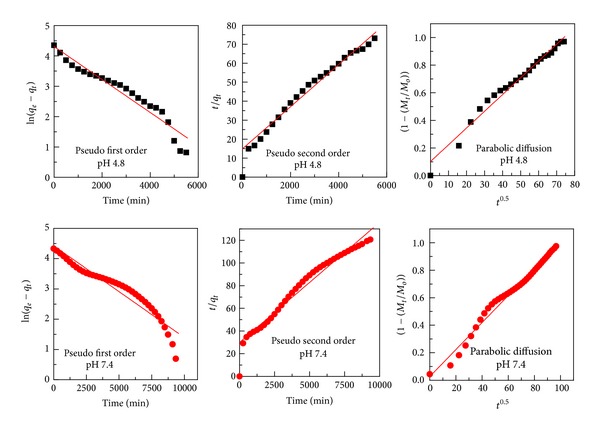
Fits for the dopa release data from Tween-dopa-LDH nanocomposite to the pseudo-first- and second-order kinetics models as well as to the parabolic diffusion model at pH 7.4 and 4.8.

By using the three kinetic models in the release kinetic data of dopa, it was found that the parabolic diffusion model and pseudo-second-order are more satisfactory for describing the release kinetic processes of dopa from the coated dopa nanocomposite at pH 7.4 and 4.8, respectively, with correlation coefficient values 0.9828 and 0.9803, respectively, (Figure 8 and Table 1). The rate constant for the parabolic diffusion model is 9.8 × 10^−3 ^min^−1^, whereas the rate constant for the pseudo-second-order was 8.2 × 10^−6 ^mg/min (Table 1).

### 3.7. Cell Viability Assessment of Tween-80 Coating on PC 12 Cells

**Figure 9:**
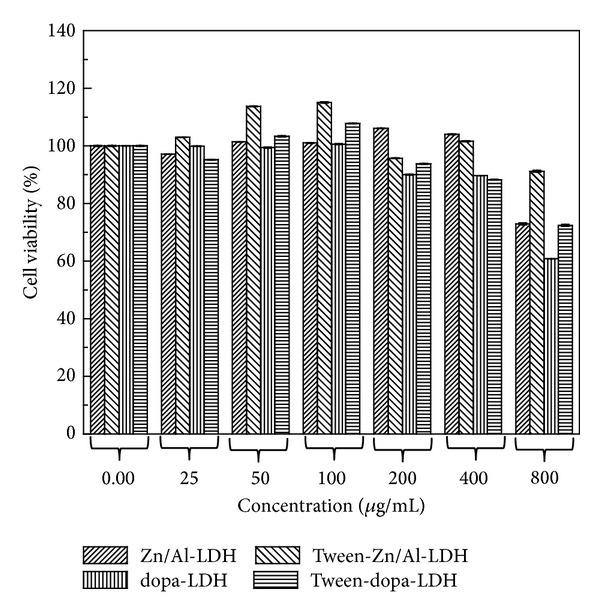
PC12 cell viability study using MTT assay. Treatment was done with the nanocomposite at doses 25–800 *μ*g/mL for 72 hours. Statistically there is no significant difference between all the groups tested (*P* > 0.05) as tested by one-way ANOVA. Tween-dopa-LDH showed slightly higher viability than dopa-LDH at 200–800 *μ*g/mL concentration. Tween-LDH also had less toxicity effect on the cell between 200–800 *μ*g/mL concentrations.

Cell viability study was done using a dopaminergic neuronal cell line PC 12 which is commonly used as* in vitro* Parkinson disease model [21, 22]. The cells were exposed to different concentrations of coated and uncoated nanocomposite (dopa-LDH, Zn/Al-LDH, tween-dopa-Zn/Al-LDH, and tween-LDH) over a period of 72 hrs, and at the end of the exposure period cell proliferation assay shows sustained cell survival even at higher doses (Figure 9). No significant cell death (cytotoxicity) was observed with all the four agents exposures within the dose range of 25–800 *μ*g/mL compared to the control (untreated wells). In most of the treatments, more than 95% of the exposed cells survived compared to control. The tween 80 coated nanocomposite (tween-dopa-LDH and tween-LDH) demonstrated higher cells survival at the highest concentration (800 *μ*g/mL) used compared to their corresponding uncoated nanocomposite (dopa-LDH and Zn/Al-LDH), respectively.

The slight difference in viability assay at higher concentration between coated and the uncoated may be the result of changes imposed by the surface coating on the nanoparticles. It was reported earlier that, physiochemical characteristics of nanoparticles like their surface charges, crystalline morphology, thermal stability, sustain drug release ability, cellular/tissue uptake, target delivery, and ultimately toxicity profile are shown to be affected with introduction of surface coating [23].

In a related study, iron oxide coated with dextran and polymer polyethylene glycol (PEG) demonstrated decrease of cytotoxicity compared to their corresponding uncoated nanoparticles; not only the toxicity but also the cell morphology were better maintained with the coated nanoparticle than the uncoated particles [24]. This result shows that our nanodelivery system containing levodopa coated with tween 80 to have similar or even better survival potential on PC 12 than the uncoated nanoparticles.

**Figure 10:**
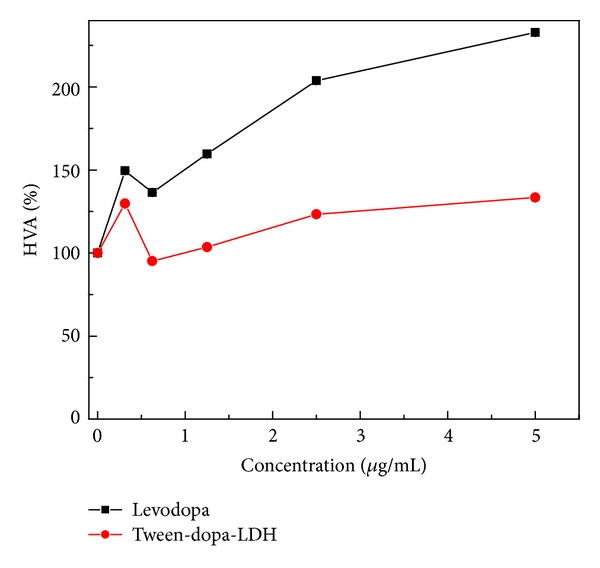
*In vitro* drug delivery and metabolism by PC 12 cells. Dopamine metabolite production from PC 12 cells 24 hours after treatment with increase concentration of levodopa and the corresponding Tween-dopa-LDH nanocomposite. HVA production increases with increase dopamine concentration from both naked levodopa and intercalated one.

Nanotechnology in drug delivery system has the advantage of targeted delivery in a sustained release fashion compared to the conventional drug delivery system [6]. HVA is a metabolite produce from dopamine metabolism at the peripheral level or in the central nervous system; in Figure 10, we use a Parkinson's disease model (PC12) to show levodopa release from zinc-aluminum nanocomposite via its metabolite compared to pure levodopa. In release study above (Figure 7), levodopa release from the coated nanocomposite (Tween-dopa-LDH) was demonstrated to be slow and sustain, that lasted several days. This may explain the lower metabolite (HVA) production by the coated nanoparticle compared to pure levodopa (Figure 10). In the case of pure levodopa, its metabolism may be rapid and immediate since almost hundred percent of it is available for metabolism after uptake by the dopaminergic cells. This experiment further strengthens sustain releasing ability of this coated nanocomposite and its possible uptake and metabolism of tween-dopa-LDH nanocomposite by the neural cells (PC 12).

## 4. Conclusion

Tween-dopa-LDH nanocomposite was prepared using Tween-80 water-soluble. The dopa-LDH nanocomposite was coated by Tween-80 in an aqueous solution. The release of levodopa from Tween-dopa-LDH nanocomposite suffered a retarding effect, which is due to coating with Tween-80. The viability study showed improved cell survival in Tween-80 coated nanocomposite than the corresponding uncoated nanocomposite. Levodopa metabolism from Tween-dopa-LDH nanocomposite possibly followed a slow and sustained release fashion demonstrated in the release study. Thus, the successful coating of depo-LDH with Tween-80 may help in delivering LDH nanocomposite to the brain via Apo lipoprotein acquisition and subsequent LDL receptor attachment on BBB surface.

## Figures and Tables

**Table 1:** Correlation coefficients (*R* ^2^) and rate constants (*k*) obtained by fitting the dopa release data for the coated nanocomposite in buffer solutions at pH 4.8 and 7.4.

pH	Saturation release (%)	*R* ^2^			Rate constant (*k*)
		Pseudo-first-order	Pseudo-second-order	Parabolic diffusion model	
7.4	78%	0.8983	0.9718	0.9828	(9.8 × 10^−3^)^a^
4.8	75%	0.9224	0.9803	0.9689	(8.2 × 10^−6^)^b^

Notes: ^a^estimated using parabolic diffusion model; ^b^estimated using pseudo-second-order kinetics.
